# Enrichment of antibiotic resistance genes within bacteriophage populations in saliva samples from individuals undergoing oral antibiotic treatments

**DOI:** 10.3389/fmicb.2022.1049110

**Published:** 2022-11-08

**Authors:** Tilde Andersson, Geofrey Makenga, Filbert Francis, Daniel T. R. Minja, Soren Overballe-Petersen, Man-Hung Eric Tang, Kurt Fuursted, Vito Baraka, Rolf Lood

**Affiliations:** ^1^Department of Clinical Sciences, Lund University, Lund, Sweden; ^2^National Institute for Medical Research, Tanga Center, Tanzania; ^3^Karolinska Institutet, Solna, Sweden; ^4^Bacterial Reference Center, Statens Serum Institute, Copenhagen, Denmark; ^5^Department of Bacteria, Parasites and Fungi, Statens Serum Institut, Copenhagen, Denmark

**Keywords:** Bacteriophage, antibiotic resistance, saliva, microbiota, antibiotic treatment

## Abstract

Spread of antibiotic resistance is a significant challenge for our modern health care system, and even more so in developing countries with higher prevalence of both infections and resistant bacteria. Faulty usage of antibiotics has been pinpointed as a driving factor in spread of resistant bacteria through selective pressure. However, horizontal gene transfer mediated through bacteriophages may also play an important role in this spread. In a cohort of Tanzanian patients suffering from bacterial infections, we demonstrate significant differences in the oral microbial diversity between infected and non-infected individuals, as well as before and after oral antibiotics treatment. Further, the resistome carried both by bacteria and bacteriophages vary significantly, with *bla*_CTX-M1_ resistance genes being mobilized and enriched within phage populations. This may impact how we consider spread of resistance in a biological context, as well in terms of treatment regimes.

## Introduction

The increased use of antimicrobials and development of resistance is at an alarming rate (Aslam et al., 2018). The emergence and spread of antimicrobial resistance has been listed by the WHO as one of the major threats to global health security (Roberts and Zembower, 2021). Specifically developing countries have high prevalence of multidrug resistant bacteria (Ayukekbong et al., 2017). Tanzania, being a developing country, has rapidly grown its economy during the last decade (Mrema et al., 2015) but has also seen a significant rise of antimicrobial resistance in particular due to the inappropriate use of antimicrobials in both the human and animal sectors (WHO Joint External Evaluation of IHR Core Capacities of the United Republic of Tanzania, 2017). Recent prevalence studies of antibiotic usage in Tanzania documented a high usage for children and patients admitted to surgical and pediatric wards (Seni et al., 2020). Such prescription was not based on culture-testing or antimicrobial susceptibility testing, but was commonly prescribed without any tests. Similarly, an excessive usage of third-generation cephalosporins (ceftriaxone) among hospitalized patients could be detected in 51.1% (322/630) individuals (Seni et al., 2020). The emergence and spread of multidrug resistant bacteria to both first- and second-line drugs (e.g., amoxicillin, chloramphenicol, trimethoprim-sulfamethoxazole, extended-spectrum cephalosporins, and fluoroquinolones) is well documented in Tanzania (Moremi et al., 2016). The rate of extended spectrum beta-lactamase (ESBL) producing *Escherichia coli* and MRSA has been increasing at an alarming rate (Moremi et al., 2014; Katale et al., 2020), with high prevalence of also other resistance genes [*bla*_CTX-Ms_ (45.7%), SCCmec type III (27.3%), IMP types (23.8%); Katale et al., 2020], with resistance to third generations cephalosporins ranging from 26 to 100% in parts of Tanzania (Masinde et al., 2009; Mushi et al., 2014; Ampaire et al., 2016; Moremi et al., 2016; Sonda et al., 2019). Likely these numbers are however too low due to lack of appropriate microbiological diagnostic capacity and infrastructure in most clinical settings, as well as inadequate resources to implement national wide surveillance programs (Katale et al., 2020).

Usage of antibiotics is however not only adding selective pressure for resistant bacteria but has also been shown to be a key factor for induction of temperate bacteriophages and spread of resistance through transduction (Stanczak-Mrozek et al., 2017). An earlier *in vivo* study in mice by Modi et al. demonstrated the impact of antibiotics at subclinical concentrations to induce bacteriophages carrying resistance genes, able to transduce sensitive bacteria (Modi et al., 2013). Similar effects have been detected in humans undergoing antibiotic treatments with increased abundance of bacteriophages carrying resistance genes (Abeles et al., 2015). However, the latter study was very limited in number of patients included (*n* = 4). There is therefore a need to investigate such putative induction in higher resolution. We recently described how the presence of specific protozoa (*Entamoeba gingivalis*) can affect the oral microbial diversity (Stensvold et al., 2021). Herein, we study alterations of microbial diversity and prevalence of antibiotic resistance carrying bacteriophages in a Tanzanian patient group undergoing antibiotic treatment.

## Materials and methods

A cross-sectional study was carried out in June 2019 at Tanga Regional Referral Hospital, Tanzania. The study was approved by the Medical Research Coordinating Committee of the National Institute for Medical Research (NIMR MRCC; reference number, NIMR/HQ/R8.a/Vol.IX/3079). Inclusion criteria for individuals entailed age (18 years or above), no usage of antibiotics during the last 3 months, a medical condition treated with oral antibiotics for at least 3 days (patients only) and with full mental capacities. Individuals were excluded from the study if they had non-infectious immune-modulating sicknesses. All individuals signed a written informed consent before enrolment in the study. The study enrolled 25 patients, who had been prescribed oral antibiotics (3–10 days) for treatment of non-oral infections, and 26 patients at the hospital who had not received antibiotics (e.g., non-infectious reasons for hospitalization for 3–10 days). Saliva samples (5 ml) were collected on day 0 (hospitalization day) and day 3. For all samples, information on age, gender, and treatment was available.

### Bacterial and bacteriophage DNA isolation

Bacterial DNA was isolated using Norgen’s Saliva DNA preservation and isolation kit (Norgen Biotek Corp., Thorold, ON, Canada). For bacteriophage DNA preparations, saliva samples were centrifuged (10,000 *g* 10 min), and the supernatant passed through sterile filters (0.22 μm) to remove bacterial contaminants. The samples were then processed using a phage DNA isolation kit (Norgen Biotek), according to manufacturer’s instructions, including addition of DNAse I and heat-inactivation thereof. DNA was quantified with Qubit fluorimeter (Life Technologies, Carlsbad, CA, United States).

### 16S/18S amplicon-based microbiome analysis

Amplicon-based microbiome analysis was done as previous described (Ring et al., 2017). Library preparation was performed by Nextera XT DNA Library Preparation (Illumina inc., San Diego, California, United States), and Illumina sequencing was performed on the Hiseq system (Illumina) according to the manufacturer’s instructions. DNA was amplified using a two-step PCR using custom 341F/806R primers targeting the V3-V4 16S regions, and three primer sets targeting the hyper-variable regions V3-V4 of the 18S rDNA gene, and amplicons were sequenced on the Illumina MiSeq (Illumina) using the V2 Reagent Kit.

### Detection of resistance genes by metagenomic long-read sequencing

DNA was prepared for sequencing using Oxford Nanopore Technologies’ Rapid PCR Barcoding Kit (SQK-RPB004) with the following modifications to the manufacturer’s instructions: Double volume of template DNA and “FRM,” as well as 25 PCR cycles instead of 14. DNA libraries were sequenced in R9.4.1 flow cells (FLO-MIN106) in a MinION (Oxford Nanopore Technology) connected to a MinIT with MinIT Release 19.12.5 (MinKNOW Core 3.6.5, Bream 4.3.16, and Guppy 3.2.10). Raw reads were basecalled with the “Fast” configuration of the algorithm. Basecalled reads were analyzed with the mapping tool KMA (K-Mer Aligner; Dillip et al., 2018) version KMA-1.2.22 with the following parameters adapted for nanopore data: “-mem_mode -mp 20 -mrs 0.0 -bcNano -and.” KMA were used for mapping against the following databases https://www.arb-silva.de/no_cache/download/archive/release_132/Exports/Archaea (16S & 18S rRNA from bacteria, archaea, and eukarya), https://www.cbs.dtu.dk/public/CGE/databases/KmerFinder/version/20190108_stable/ (Fungi, plasmids, and protozoa), https://www.cbs.dtu.dk/public/CGE/databases/KVIT/version/20190513/ (viruses), as well as ResFinder database (2020-04-08) and PlasmidFinder (2020-04-02).

Dehumanization of the raw reads was performed using Minimap2 against the hg38 human genome reference. Reads unmapped to the human genome were extracted using samtools 1.9 with parameter -f4. All data were uploaded to the ENA browser under accession PRJEB55897 (primary) and ERP140841 (secondary), and can be found in Supplementary File 1.

### Bioinformatics analysis of sequence data

Bioinformatics was done using BION,^1^ a newly developed analytical semi-commercial open-source package for 16S rRNA and other reference gene analysis, classifying mostly to species level. The pipeline accepts raw sequence and includes steps for de-multiplexing, primer-extraction, sampling, sequence- and quality-based trimming and filtering, de-replication, clustering, chimera-checking, reference data similarities, and taxonomic mapping and formatting. Non-overlapping paired reads are allowed for analysis, and BION is often accurate to the species level.

### Statistics of sequence data

Analysis of microbiome composition was performed in R version 4.0.32020-10-10 using the packages phyloseq v.1.24.2 and vegan v.2.5-2. Figures were created using ggplot2 v.3.2.0 and plotly v.4.8.0. In the bar plot, taxa were merged to genus level by agglomerating counts within each genus. Alpha-diversity of samples as well as relative abundances of individual genera were compared between groups with Mann–Whitney rank sum tests and adjusted for multiple testing using Bonferroni correction. Differences between groups were assessed with bar plots and Principal Coordinates Analysis (PCoA) plots using Bray-Curtis distances and tested with a permutational multivariate ANOVA (PERMANOVA), “*adonis*” from the package “*vegan*” v.2.5-2.

### ddPCR amplification of resistance genes

A PCR reaction was prepared according to BioRad’s 2xddPCR Supermix for probes (no dUTP) instructions (BioRad). Droplets were prepared and analyzed in a QX200 ddPCR system and evaluated with quantasoft 1.7. The PCR reaction was conducted in a BioRad C1000 thermal cycler, following standard cycling settings. For bacteriophage DNA, a ddPCR targeting 16S was routinely included to confirm a low or absent presence of contaminating bacterial DNA. Prevalence of <100 gene copies of 16S per 6 ng viral DNA was deemed acceptable. Primers used for the reactions can be found in Table 1.

**Table 1:** ddPCR-primers used in the study.

Target	Forward primer	Reverse primer	Probe
16S	AGAGTTTGATCCTGGCTCAGGA	CGTGTTACTCACCCGTCCG	CGCTGGCGGCGTGCCTAATACATGC
*bla*_CTX-M1_	ACAGTACAGCGATAACGTGG	GAATGGCGGTGTTTAACGTC	GCGGCCCGGCTAGCGTCACC
*bla*_OXA-48_	AAGTTACACGTATCGGAGCG	ACCAGCCAATCTTAGGTTCG	AGCCATGCTGACCGAAGCCAATGGTGA
*tetA*	TTGAACGGCCTCAATTTCCT	GATGAAGAAGACCGCCATCA	GCATGACCGTCGTCGCCGCCC
*qnrS1*	GGGTGCATCACTGAAAGAGT	CCAGTGCTTCGAGAATCAGT	TGCCACGCCGAACTCGACGGTTTAGA

## Results

### Individuals with ongoing infection differs in microbial composition in the saliva

In total, 51 individuals visiting the hospital due to infection [*n* = 25, patient *d*(0)] or due to non-infectious illness [*n* = 26, control *d*(0)] were enrolled in the study and DNA from saliva isolated. The patients were treated for a diversity of infections, including urinary tract infections, otitis media, pelvic inflammatory disease, gastritis, pneumoniae, and pyomyositis. Three days after initial check-up, individuals were tested again for possible changes in the microflora (d3). Four individuals in the infection group did not receive antibiotics, and additionally five individuals from that group did not leave a second sample. One individual from the control group failed to leave a follow-up sample on day 3. Patient information can be found in Table 2.

**Table 2:** Patient information.

Group	*n*	Gender (f/m)	Age (mean, range; median)
Patient (d0)	25	17/8	37 (18–83); 30
Control (d(0))	26	11/15	27 (20–43); 25
Patient (d3; tot)	16	12/4	41 (20–83); 36
Patient (d3; +)	12	8/4	43 (20–83); 36
Patient (d3; −)	4	4/0	36 (23–54); 33
Control (d3)	25	10/15	27 (20–43); 25

**Figure 1:**
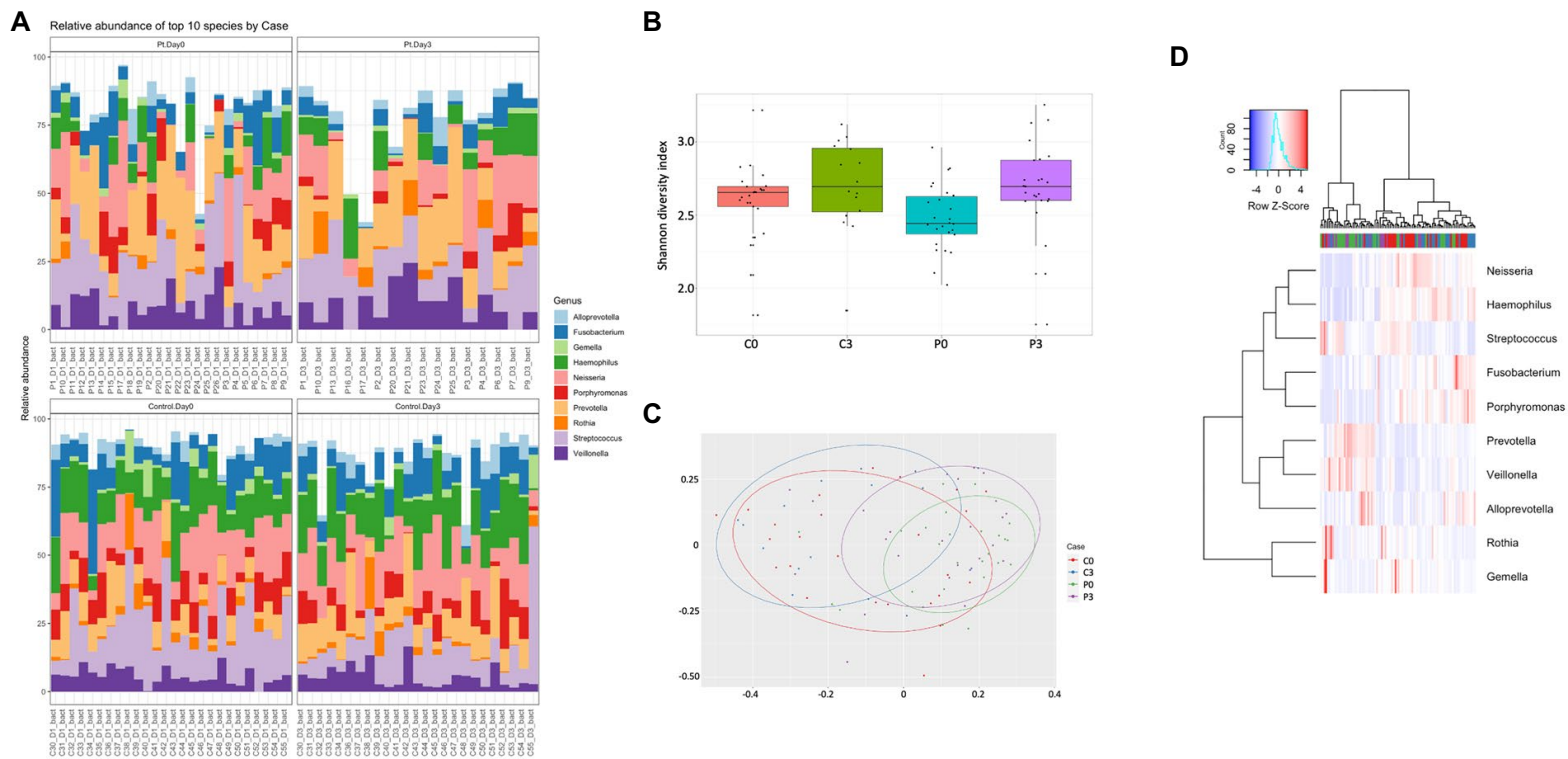
Individuals with ongoing infection have altered prokaryotic compositions in the saliva. The prokaryotic composition from the cellular fraction of individuals undergoing oral antibiotic treatment was compared with a control group. The main identified genus among bacteria **(A)** are reported, together with alpha **(B)** and beta **(C)** diversity. A heat map summarizing the findings can also be visualized in **(D)**.

The microbiome was significantly different, with control individuals having less prokaryotic diversity, both in alpha and beta (Figures 1B,C) compared to the patients undergoing antibiotic treatment. The control group had minor, but significant, changes in the flora over the course of the analysis, in particular in *Selenomas* (*p* = 0.001), *Streptococcus* (*p* = 0.002), and *Propionibacterium* (*p* = 0.02). Similarly, the patient group had minor changes over the course of the analysis, in particular in *Propionibacterium* (*p* = 0.005) and several species of *Clostridiales* (*p* = 0.006–0.008; Figures 1A,D). More significant differences could be detected in patients vs. controls, in particular regarding *Selenomas* (*p* = 0.00007, day 0), *Haemophilus* (*p* = 0.0001 day 0, *p* = 0.0008 day 3), *Actinomyces* (*p* = 0.0005 day 0), *Prevotella* (*p* = 0.0006 day 0), *Fusobacteria* (*p* = 0.002 day 3), *Klebsiella* (*p* = 0.01 day 3), and *Porphyromonas* (*p* = 0.02 day 3), a difference that persisted even after antibiotics treatment (Figures 1A,D).

**Figure 2:**
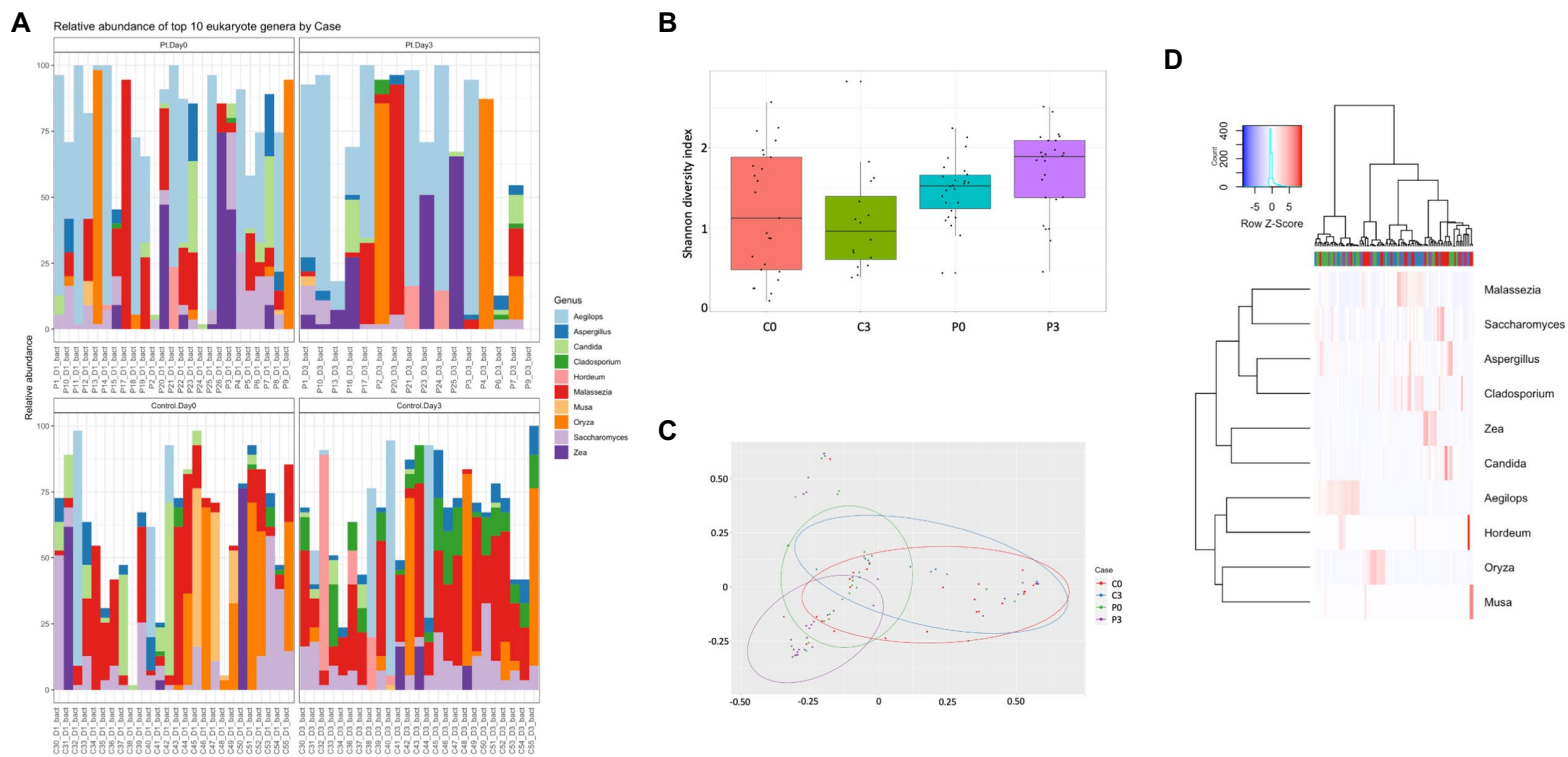
Individuals with ongoing infection have altered eukaryotic compositions in the saliva. The eukaryotic composition from the cellular fraction of individuals undergoing oral antibiotic treatment was compared with a control group. The main identified genus among eukaryotes **(A)** are reported, together with alpha **(B)** and beta **(C)** diversity. A heat map summarizing the findings can also be visualized in **(D)**.

For the eukaryotic composition, there were similarly only minor changes over time, with only *Cladosporium* (*p* = 0.00001) and *Oenothera* (*p* = 0.012) being affected in the control group. No significant changes could be detected in the patient group over time, however, in relation to the control group, several eukaryotes displayed significant changes in prevalence, including *Aegilops* (*p* = 0.0002, day 0, *p* = 0.013 day 3), *Batcheloromyces* (*p* = 0.009 day 0), *Oenothera* (*p* = 0.012 day 0), *Cladisporium* (*p* = 0.0001 day 3), *Malassezia* (*p* = 0.0014 day 3), and *Saccharomyces* (*p* = 0.0016 day 3), Figure 2.

**Figure 3:**
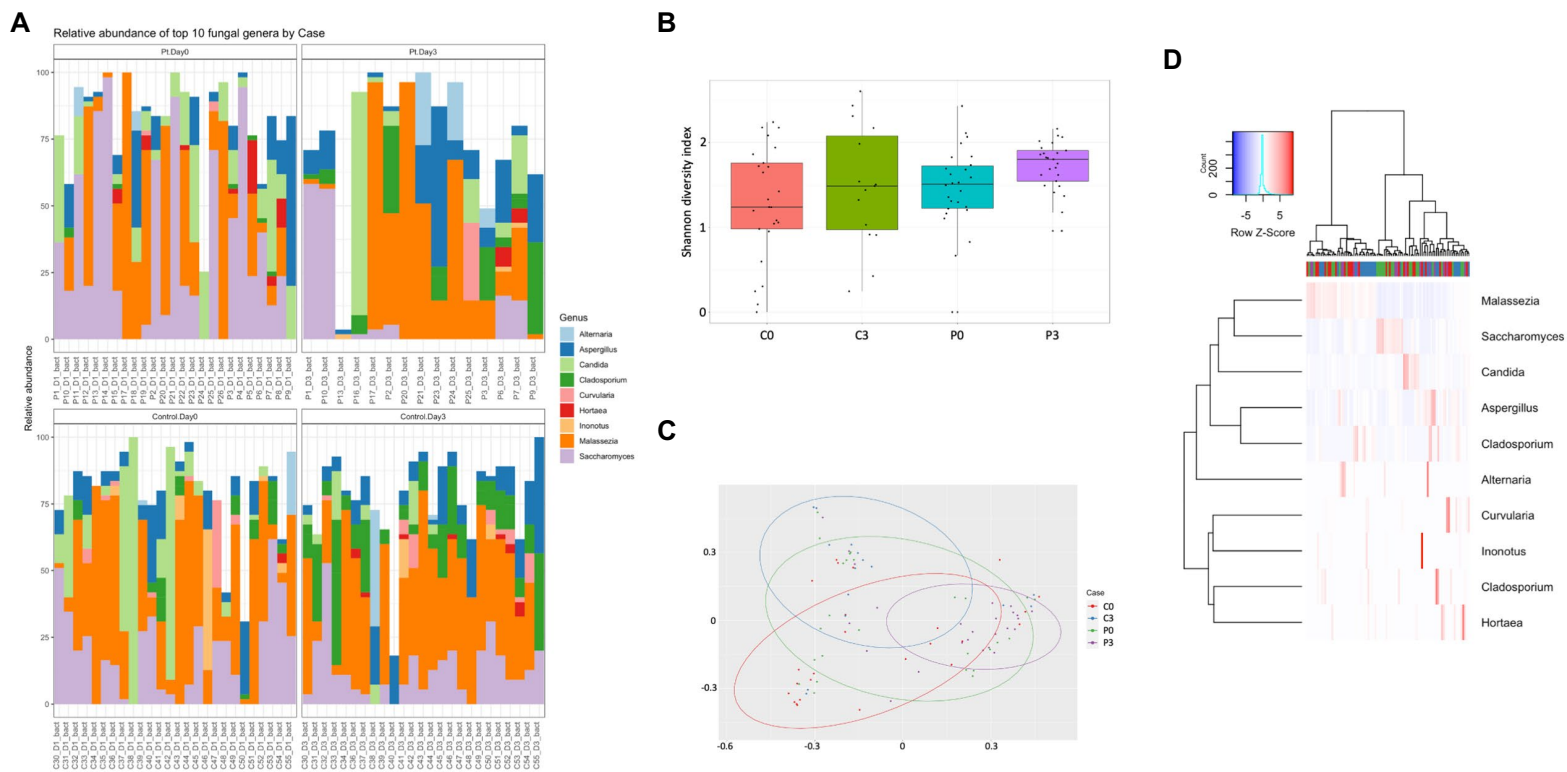
Individuals with ongoing infection have altered fungal compositions in the saliva. The fungal composition from the cellular fraction of individuals undergoing oral antibiotic treatment was compared with a control group. The main identified genus among fungi **(A)** are reported, together with alpha **(B)** and beta **(C)** diversity. A heat map summarizing the findings can also be visualized in **(D)**.

Looking further into the eukaryotes, focusing on the fungal population, some time-resolved differences could be detected of significance, both within the patient group (*Cladosporium, p* = 0.0007; *Saccharomyces, p* = 0.005; and *Inonotus*, *p* = 0.024) and within the control group (*Cladosporium, p* < 0.00001; *Hortaea, p* = 0.02; and *Ustilago*, *p* = 0.019). A significant difference could also be detected between the control group and the patient group at the start of the antibiotic treatment (*Geotrichum, p* = 0.009; *Batcheloromyces, p* = 0.009; *Hortaea*, *p* = 0.019; and *Inonotus, p* = 0.024); a significant that was reduced after 3 days of antibiotic treatment, where only *Saccharomyces* was significantly different (*p* = 0.025), Figure 3.

### Bacteriophage fractions from patients have a lower diversity than control individuals

**Figure 4:**
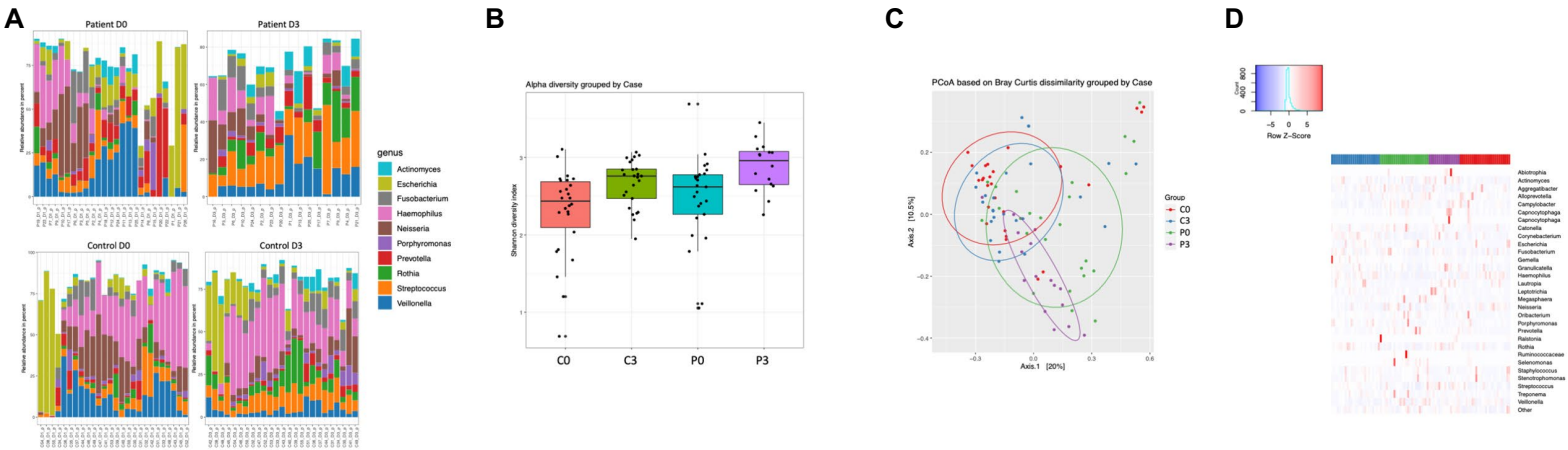
Bacteriophage genetic diversity is linked to patient health status. Bacteriophage DNA was isolated from saliva from patients or controls, and the microbiome and mycobiome determined through 16S and 18S amplicon sequencing. The main identified genus among bacteria **(A)** are reported, together with alpha **(B)** and beta **(C)** diversity of bacteria and. A heat map summarizing the findings can also be visualized in **(D)**.

Bacteriophages carrying bacterial DNA could be identified among both the control group and the patients, with a significant difference in beta diversity (Figure 4C; *p* = 0.001). Both groups are significantly affected over time in alpha diversity (controls: *p* = 0.008, patients: *p* = 0.007; Figure 4B). No significant difference in the alpha diversity can be detected between the two groups at the start of the treatment (*p* = 0.17); however, at the end of the treatment there is a minor significant difference (*p* = 0.04).

The variation in prokaryotic content carried by the bacteriophage particles were highly diverse, and differed significantly both over time within both the control group and the patient group, as well as between the two treatment groups (Figures 4A,D). Specifically, *Selenomas* (*p* = 0.0002), *Haemophilus* (*p* = 0.0007), *Prevotella* (*p* = 0.0016), *Clostridia* (*p* = 0.0017), and *Kingella* (*p* = 0.013) differed between the patient group and the control group before initiation of treatment, while it was significantly changed during the course of the treatment (*Haemophilus p* = 0.0002; *Streptococcus p* = 0.002; *Veillonella p* = 0.004; *Escherichia p* = 0.008; *Capnocytophaga p* = 0.008; *Staphylococcus p* = 0.013; and *Porphyromonas p* = 0.013). Similarly, the patient group had a significant change in genetic material carried by bacteriophages during the course of the treatment, in particular for *Granulicatella* (*p* = 0.0002), *Propionibacterium* (*p* = 0.0002), *Streptococcus* (*p* = 0.0002), *Leptotrichia* (*p* = 0.0003), *Rothia* (*p* = 0.0006), *Escherichia* (*p* = 0.001), *Corynebacterium* (*p* = 0.0028), and *Actinomyces* (*p* = 0.015). For the control group, a similarly vast, though different composition, was affected during the course of time: *Oribacterium* (*p* = 0.0002), *Actinomyces* (*p* = 0.0002), *Gemella* (*p* = 0.0002), *Streptococcus* (*p* = 0.0004), *Propionibacterium* (*p* = 0.0005), *Enterococcus* (*p* = 0.0009), *Lactococcus* (*p* = 0.004), *Peptostreptococcus* (*p* = 0.025), and *Pseudomonas* (*p* = 0.026). Due to the low amount of data from the mycobiome, it was not of relevance to describe species differences within this population.

### Metagenomic analysis of resistome

To scout for resistance genes within the patient group, material from one randomly selected patient (day 0 and day 3; bacterial and phage fraction) was investigated using a metagenomic analysis on the resistome. The results are summarized in Table 3.

**Table 3:** Time-resolved metagenomics of an individual patient.

Sample	ENA sample accession and run accession	Antibiotic group	Resistance genes	Virus
P (d0; bact)	SAMEA111353510 ERR10188468	Tetracycline macrolide betalactam phenicol aminoglycoside sulphonamide	*tet*(A, B, M, Q, W) *erm*(B, F), *mef*(A), *mphE*, *msr*(D) *penA*, *bla*_OXA-85_, *cfx*(A3, A5) *catQ*, *cat*(pC194) *Isa*(C), and *aph*(3′)-Ia *sul2*	Human endogenous retrovirus K113, K115, and HCML-ARV
P (d0; phage)	SAMEA111353511 ERR10188469	Tetracycline betalactam aminoglycoside	*tet*(C) *bla*_TEM-1A_, 231, *bla*_OXA-22_, and 60 *aph*(3′)-Ia	Human endogenous retrovirus HCML-ARV
P (d3; bact)	SAMEA111353512 ERR10188470	Tetracycline macrolide betalactam phenicol aminoglycoside sulphonamide	*tet*(A, B, M, O, Q, W), *erm*(B, F), *med*(A), *mphE*, *msr*(D) *penA*, *bla*_OXA-85_, *cfx*(A2, A5) *catQ*, *cat*(pC194) *Isa*(C), *aph*(3′)-Ib, and *aph*(6)-Id *sul2*	Human endogenous retrovirus K113, K115, and HCML-ARV
P (d3; phage)	SAMEA111353513 ERR10188471	Tetracycline macrolide	*tet*(M, O, W) *mef*(A), *msr*(D), and *erm*(F)	Human endogenous retrovirus K113, K115, and HCML-ARV

### Prevalence of resistance genes is dependent on infection and gender

**Figure 5:**
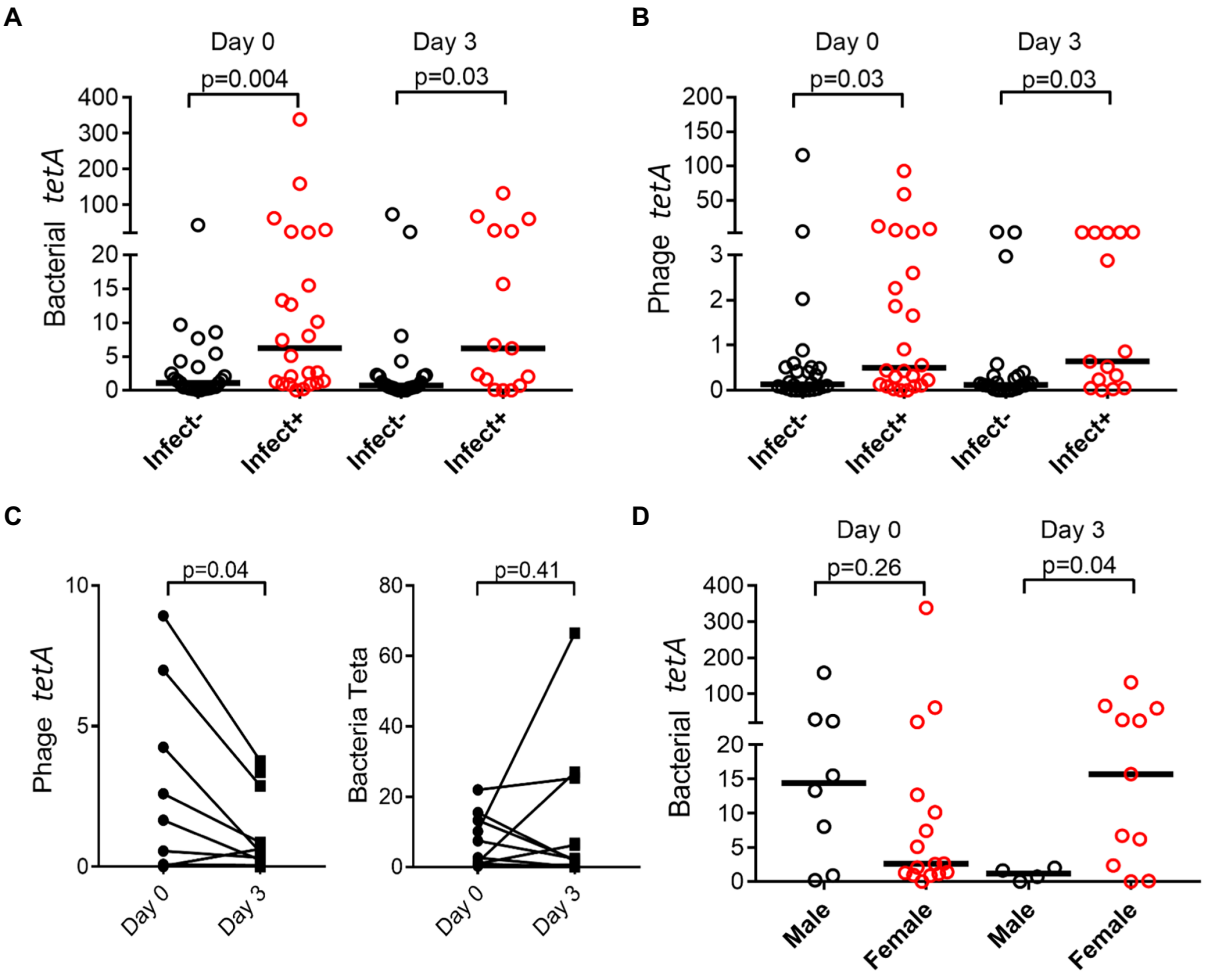
Prevalence of resistance genes is dependent on infection and gender. *tetA* was quantified by ddPCR, and abundance compared between patients (Infect+) and controls (Infect−) change over time in bacterial **(A)** and phage fraction **(B)**. Effect on antibiotic usage on *tetA* prevalence in bacterial and phage fractions was also evaluated **(C)**, as well as if gender affected prevalence **(D)**. The *y*-axis represents relative number of gene copies in the sample.

Both in the bacterial and bacteriophage population, carriage of resistance genes was significantly higher in individuals with infections, both in bacterial and phage fractions (Figures 5A,B). The abundance of resistance genes was not affected over time (e.g., 3 days), however, a gender aspect could be detected with significantly more women than men having remaining high levels of *tetA* after antibiotic treatment (Figure 5D). Prevalence of resistance toward tetracycline (*tetA*) remained unaffected in the bacterial population but did however decrease significantly in the phage population (Figure 5C).

### Resistance genes are differently associated with bacterial and bacteriophage populations

**Figure 6:**
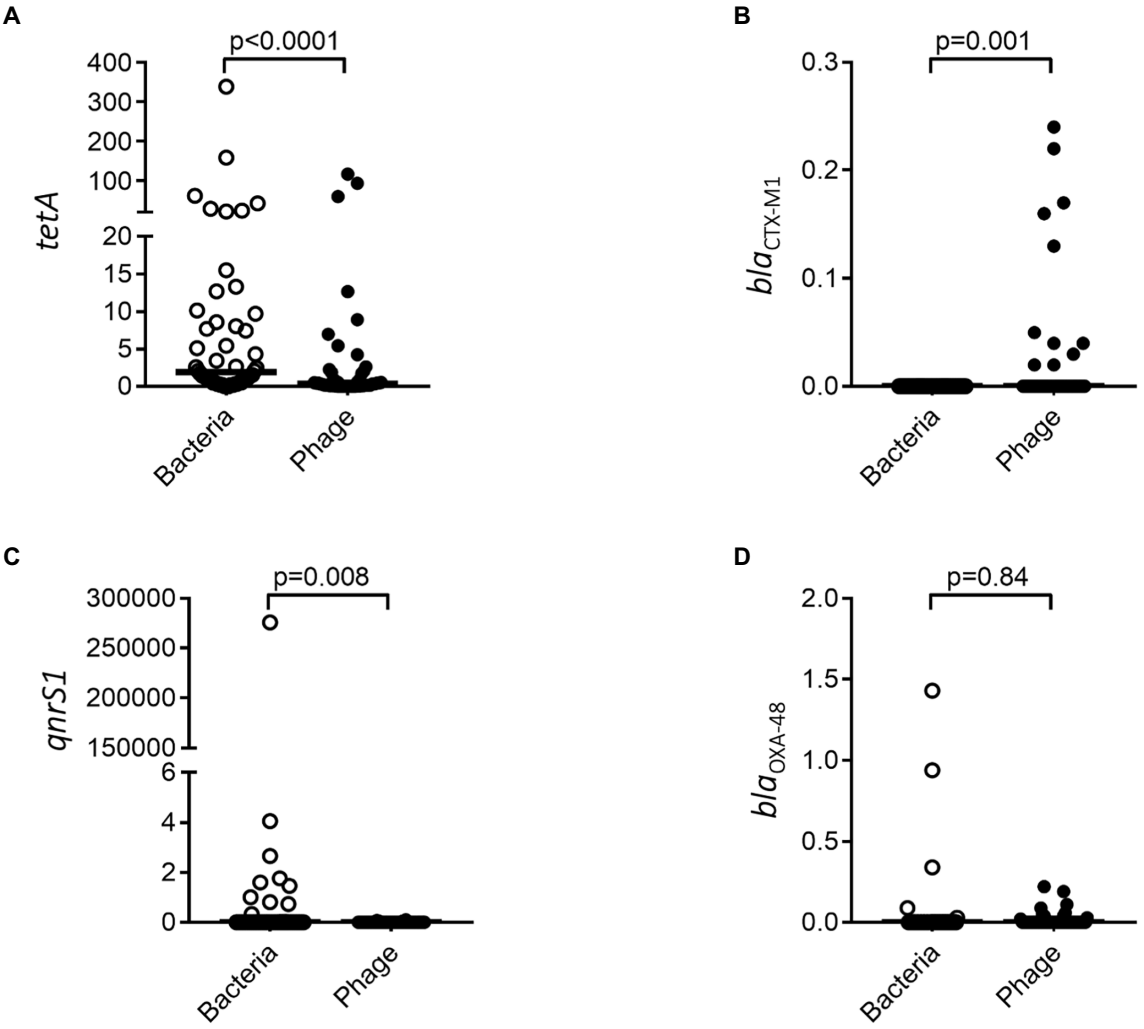
Resistance genes are differently associated with bacterial and bacteriophage populations. *tetA* **(A)**, *bla*_CTX-M1_ **(B)**, *qnrS1* **(C)**, and *bla*_OXA-48_ **(D)** were quantified by ddPCR for both the bacterial and bacteriophage population. The *y*-axis represents relative number of gene copies in the sample.

Having demonstrated that resistance genes could vary between the bacterial and phage population over time, we investigated the abundance of resistance genes toward four common antibiotics in both populations. *tetA* and *qnrS1* resistance was significantly more abundant in the bacterial fraction (Figures 6A,C) while *bla*_OXA-48_ did not show any significant difference (Figure 6D). *bla*_CTX-M1_ could only be identified in the bacteriophage fractions (Figure 6B) where it was enriched as compared to the bacterial fraction.

## Discussion

The ability of bacteriophages to transduce genes has been known for soon 70 years; however, its importance in horizontal gene transfer has often been overshadowed by conjugation due to its higher efficiency. Recent reports have however demonstrated how bacteriophages can act as reservoirs of both resistance and virulence genes (Modi et al., 2013), and as such serve a complementary role to conjugation in spread of resistance. In particular this is of importance in environments exposed to bacteriophage stressors, inducing bacteriophages enabling them to transduce more efficiently. Such stressors can include sub-clinical levels of residual antibiotics, that while unable to kill bacteria still can exert a negative stress upon the bacteria leading to phage induction (Goerke et al., 2006; Torres-Barceló, 2018). Indeed, a notorious hotspot for transduction events is wastewater treatment plants, where high concentrations of microorganisms can be found together with several stressors (Rizzo et al., 2013; Lood et al., 2017). In such milieus, bacteriophage induction is a potent mechanism for horizontal gene transfer and organic material from WWTPs has been demonstrated to facilitate resistance spread through bacteriophages in soil (Gatica and Cytryn, 2013; Marano et al., 2021). Most wastewater treatment plants have only been designed for the removal of resistant bacteria, not specifically resistant genes encapsulated within bacteriophages, hence allowing for such resistance spread even within developed countries. It is however not only in the environment and within our wastewater that resistance spread can be facilitated through bacteriophages. Modi et al. brilliantly demonstrated for the first time 2013 how mice fed with subclinical levels of antibiotics induced fecal bacteriophages, carrying resistance, and virulence genes acting as a reservoir for sensitive bacteria that could be transduced into a recipient microflora (Modi et al., 2013). Importantly, the composition of resistance genes carried within the phageome was highly dependent on the type of stress, indicating that choice of antibiotics for treatment of infections may be highly relevant for limiting spread of resistance through bacteriophages (Modi et al., 2013). Abeles et al. followed up the findings by Modi, moving from a highly controlled mouse experiment to a human setting (Abeles et al., 2015). Despite their low amount of participants in the study (*n* = 4 patients, *n* = 5 controls), they could detect a significant increase in the fecal resistome. Such increase could not be detected in the oral cavity. With the antibiotics only being present in the oral cavity for a short time span, a significant upregulation of phages may not necessarily be expected, however, a significant change in the virome itself could be detected (Abeles et al., 2015), indicating that a larger study may be able to identify such induction. Having demonstrated that stressors in environmental water can affect prevalence of resistance genes and carriage by bacteriophages (Andersson et al., 2022), we thus set out to investigate if phage induction would take place in patients receiving oral antibiotics, and how this would affect the resistance profile.

Health has been strongly associated with a highly diversified microbiota, with lowered diversity associated with increased risks of several important diseases, including autoimmune diseases, heart-related diseases, and cancer (Manor et al., 2020). The composition of the microbiota has also been indicated to play a pivotal role in our immune defense against pathogens (van Rensburg et al., 2015). In our material, patients had a higher diversity of microbes compared to controls, as well as an altered composition. The material is however too small to make any claims in regard to associations between specific genus of bacteria and health. Similar to the diversity within the cellular material, purified genetic content from the bacteriophage population demonstrated significant differences between the two groups.

Due to the generally lower amount of phage DNA isolated from the samples, as compared to bacterial DNA, a common complication is significant levels of contaminating bacterial DNA in the material (Göller et al., 2020). Such contamination may mask biological changes within the phage fraction or lead to misinterpretations of the results. Importantly, we did not detect any major general carry-over of contaminating DNA from the bacterial fractions to the phage fraction (data not shown), also indicated by the different enrichments of genetic material within both the bacterial and phage fraction. A higher diversity was identified within the phage population and more significant differences between the patient group and the controls, as well as within the same group over time as compared to the prokaryotes. Such difference is however expected to be identified, due to the higher individual specificity and flexibility of the phageome (Shkoporov et al., 2018). Further, we detected a discrepancy in the distribution of antibiotic resistance genes. Overall, resistance genes were more commonly identified within individuals having ongoing infections, both in the phage and bacterial fraction, which is expected due to the higher abundance of bacteria as well as selective pressure during infections. We could however not demonstrate an antibiotic triggered phage induction or increased phage carriage of resistance genes in the saliva, in line with Abeles et al. (2015), despite having a larger patient material. Possibly, phages induced by antibiotics will quickly be transported down to the GI tract and eventually end up in a fecal fraction, explaining why the two studies from Abeles and Modi both only found a significant increase of resistance-carrying phages in this compartment (Modi et al., 2013; Abeles et al., 2015). It should however be emphasized that the current study only demonstrates presence of resistance genes within the phage capsid, and makes no distinction between if the resistance genes are carried on the bacteriophage chromosome, or have been packaged as individual plasmids. This is particularly important since many of the affected resistance genes have not been detected integrated in bacteriophages before, but only seen as free plasmids (Cantón et al., 2012).

Though not being able to demonstrate induction of phages, we could detect changes in the phageome resistance genes during treatment, while the bacterial resistance profile remained stable over the course of treatment. Specifically, we observed a drop in resistance genes for *tetA* within the phage community. While initially this may be interpreted as opposite to our hypothesis (e.g., reduced phage induction based on antibiotic treatment), it should be noted that we only targeted four specific antibiotics, and a broader screening may be needed for a more conclusive picture. Variance in resistance gene abundance in the phage population is however in concordance with the metagenomics data from a single patient, demonstrating that in general the bacterial resistome is unaffected by the treatment (e.g., no significant difference between day 0 and day 3) while the phage resistome composition is altered. *tetC* is downregulated in favor of other *tet* variants. Furthermore, no longer can any β-lactamase or aminoglycoside resistance genes be detected; instead, a higher proportion of macrolide resistance genes encoding efflux pumps could be demonstrated. Considering the patient was given cotrimoxazole (combination of sulfonamide and dihydrofolate reductase inhibitor), a general mechanism such as efflux pumps is a potent resistance mechanism that sensitive bacteria can benefit from. Thus, while some resistance genes are less abundant in the phage fraction upon treatment with antibiotics, others will increase. Regardless of antibiotic treatment or time, we could demonstrate that both bacterial and bacteriophage fractions carry resistance genes, being distinctly enriched in the two populations. Carriage of resistance genes within phage particles has been demonstrated to be induced after induction of the SOS-response, e.g., after usage of antibiotics (Modi et al., 2013). Our data did not support such induction, however, it should be noted that the induction seen in the study by Modi et al. was performed in uninfected and otherwise unstimulated mice. Thus, within individuals with ongoing infections, bacteria may be exposed to stress spontaneously triggering induction of bacteriophages carrying resistance genes (Broudy et al., 2001).

The study setting in Tanzania allowed for a high abundance of resistance genes within the population (Mikomangwa et al., 2020), enabling us to include fewer patients. However, it also makes downstream analyses more complex in terms of molecular mechanisms, with resistance gene abundancies being affected differently by distinct antibiotics and stressors. Further, none of the patients were hospitalized, meaning that dietary effects and hygiene routines are not standardized and may differ between the individuals. Still, in the control group, we could only detect minor difference in the oral microbiota between day 0 and day 3, indicating that these factors were not highly influential in this study. Further, age and gender distribution are critical factors for microbial compositions (Camarinha-Silva et al., 2014), and thus significant differences in group compositions can affect the results. In our material, there was no significant difference between the control group and the patient group in relation to age (*p* = 0.13) or gender (*p* = 0.10).

Finally, we could demonstrate a significant impact on gender for resistance carriage, with more women carrying resistance genes after antibiotic treatment. It has been reported that women are more prone to visit hospitals and primary care centers upon illness mostly due to cultural and societal factors rather than biological (Thompson et al., 2016). Similar findings have been found in Tanzania, with a higher proportion of women attending primary health care centers as compared to men (Mboera et al., 2018). Having more traditional gender roles, several studies have shown inequalities between men and women in Tanzania (Palermo et al., 2020), with women mainly responsible for housekeeping (Clausen et al., 2018) but also including childcare and visits to hospitals (Feinstein et al., 2011). Men attending health care in Africa had a higher mortality rate due to only going there when complications already have set in, not as a preventive mean, thus being more difficult to treat (Arodiwe et al., 2014). However, studies have also indicated that for specific infections, women are reported to have significantly higher levels of resistant bacteria (Buie et al., 2004). The authors speculate that the increased incidence of resistant bacteria among women may be due to their higher visit-rate at hospitals or primary care centers (as non-infected visitors), where resistant bacteria are prevalent and upon which they have a higher likelihood of being infecting by resistant bacteria.

This study thus demonstrates how antibiotics are drivers of resistance spread through activation of bacteriophages *in vivo,* and that such resistance genes can be enriched within bacteriophages in a specific manner, serving as a resistome reservoir. This may impact how we consider spread of resistance in a biological context, as well in terms of treatment regimes.

## Data availability statement

The data presented in the study are deposited in the European Nucleotide Archive (ENA) repository, accession number PRJEB55897.

## Ethics statement

The studies involving human participants were reviewed and approved by Medical Research Coordinating Committee of the National Institute for Medical Research (NIMR MRCC) Reference number: NIMR/HQ/R8.a/Vol.IX/3079. The patients/participants provided their written informed consent to participate in this study.

## Author contributions

RL, TA, KF, and VB designed the study. TA, GM, FF, DM, SO-P, M-HT, KF, VB, and RL contributed in collecting and analyzing the data. RL wrote the main manuscript text. All authors contributed to the article and approved the submitted version.

## Funding

This work was supported by grants from The Swedish Research Council Formas (2017-00100), and Innovation Fund Denmark (7044-00005B) as part of the Joint Programming Initiative on Antimicrobial Resistance (JPIAMR) call “*Transmission Dynamics*,” and Eurofins Foundation.

## Conflict of interest

The authors declare that the research was conducted in the absence of any commercial or financial relationships that could be construed as a potential conflict of interest.

## Publisher’s note

All claims expressed in this article are solely those of the authors and do not necessarily represent those of their affiliated organizations, or those of the publisher, the editors and the reviewers. Any product that may be evaluated in this article, or claim that may be made by its manufacturer, is not guaranteed or endorsed by the publisher.

## Footnotes

^1^ https://tinyurl.com/3fn8hvaj
